# Melatonin induces progesterone production in human granulosa-lutein cells through upregulation of StAR expression

**DOI:** 10.18632/aging.102367

**Published:** 2019-10-16

**Authors:** Lanlan Fang, Yiran Li, Sijia Wang, Yiping Yu, Yuxi Li, Yanjie Guo, Yang Yan, Ying-Pu Sun

**Affiliations:** 1 Center for Reproductive Medicine, Henan Key Laboratory of Reproduction and Genetics, The First Affiliated Hospital of Zhengzhou University, Zhengzhou 450052, China

**Keywords:** melatonin, StAR, steroidogenesis, granulosa cells

## Abstract

Steroidogenic acute regulatory protein (StAR) mediates the rate-limiting step in ovarian steroidogenesis and progesterone (P4) synthesis. Melatonin and its receptors are expressed in human granulosa cells, and have been shown to influence basal P4 production. However, previous studies addressing the regulation of StAR expression by melatonin and its impact on P4 secretion yielded contradictory results. Here, we demonstrate that melatonin upregulates StAR expression in primary cultures of human granulosa-lutein (hGL) cells obtained from women undergoing *in vitro* fertilization (IVF). Using pharmacological inhibitors, we show that the stimulatory effect of melatonin on StAR expression is mediated via both MT1 and MT2 melatonin receptors. Melatonin exposure activates the PI3K/AKT signaling pathway and its inhibition attenuates the stimulatory effect of melatonin on StAR expression. Moreover, siRNA-mediated knockdown of StAR abolishes melatonin-induced P4 production. Importantly, clinical analyses demonstrate that melatonin levels in human follicular fluid are positively correlated with P4 levels in serum. By illustrating the potential physiological role of melatonin in the regulation of StAR expression and P4 production in hGL cells, our results may serve to improve current strategies used to treat clinical infertility.

## INTRODUCTION

Human reproduction is profoundly influenced by age [1]. After ovulation, ovarian granulosa cells transform into granulosa-lutein (hGL) cells that produce progesterone (P4), an essential steroid hormone that regulates luteinization and maintains the early stages of pregnancy. In patients undergoing *in vitro* fertilization (IVF), premature luteinization is defined as an increase in serum P4 levels before or on the day of human chorionic gonadotropin (hCG) administration. Several studies have demonstrated that premature luteinization is associated with decreased implantation and pregnancy rates [2, 3]. In contrast, insufficient ovarian P4 production (i.e. luteal phase deficiency) is associated with dysfunction of the secretory endometrium, which compromises successful embryo implantation and growth [4]. Therefore, a precise regulation of P4 secretion in hGL cells is required to maintain normal reproductive functions.

Although pituitary luteinizing hormone (LH) plays a central role in the induction of P4 secretion in the ovary, accumulating evidence suggests that P4 biosynthesis can also be regulated by locally-produced factors that exert their effects in an autocrine and/or paracrine fashion [5, 6]. Melatonin, a pineal hormone, regulates major physiological functions including the sleep-wake cycle, pubertal development, and seasonal adaptation [7]. While most endogenous melatonin is synthesized and released at night by the pineal gland, this hormone is also produced by extra-pineal organs such as the ovary, where it was shown to regulate reproductive functions through both receptor-mediated signaling affecting cellular metabolism, and receptor-independent actions as a scavenger for reactive oxygen and nitrogen species [8–10]. Research has shown that melatonin levels in serum are reduced with aging [9, 11], potentially impacting reproductive potential in women. Melatonin acts on target cells by binding to and activating two membrane-bound G-protein-coupled receptors, MT1 (*MTNR1A*) and MT2 (*MTNR1B*) [12], both of which are expressed in hGL cells [16]. Interestingly, melatonin expression can be detected in human follicular fluid at higher concentrations than those present in serum [13–15], suggesting its relevance in the regulation of follicular function.

Steroidogenesis is a complex process that involves multiple enzymatic reactions [17]. P4 is initially synthesized from cholesterol in the mitochondria. Once free cholesterol has been transported to the mitochondria, it is transferred from the outer to the inner mitochondrial membrane by steroidogenic acute regulatory protein (StAR). This transfer represents the rate-limiting step of steroidogenesis and P4 production in granulosa cells [18–20]. Previous studies on different animal models have shown that exogenous melatonin can stimulate the production of P4 by granulosa cells [21–23]. Although various factors and signaling pathways are reported to regulate StAR expression [24], to date only a handful of animal studies have examined the effects of melatonin on StAR expression in the ovary [25–27]. Meanwhile, in hGL cells the effect of melatonin on basal P4 production remains controversial [28].

Therefore, through pharmacological inhibition of melatonin receptors, siRNA-mediated knockdown of StAR, and clinical measurements of follicular melatonin and serum P4 levels, the present study reveals a dose- and time-dependent stimulatory effect of melatonin on both StAR expression and P4 production in cultured hGL cells. Our results highlight a potential physiological mechanism by which melatonin influences ovarian steroidogenesis and might help design new approaches for the treatment of clinical infertility.

## RESULTS

### Melatonin stimulates StAR expression in hGL cells

**Figure 1:**
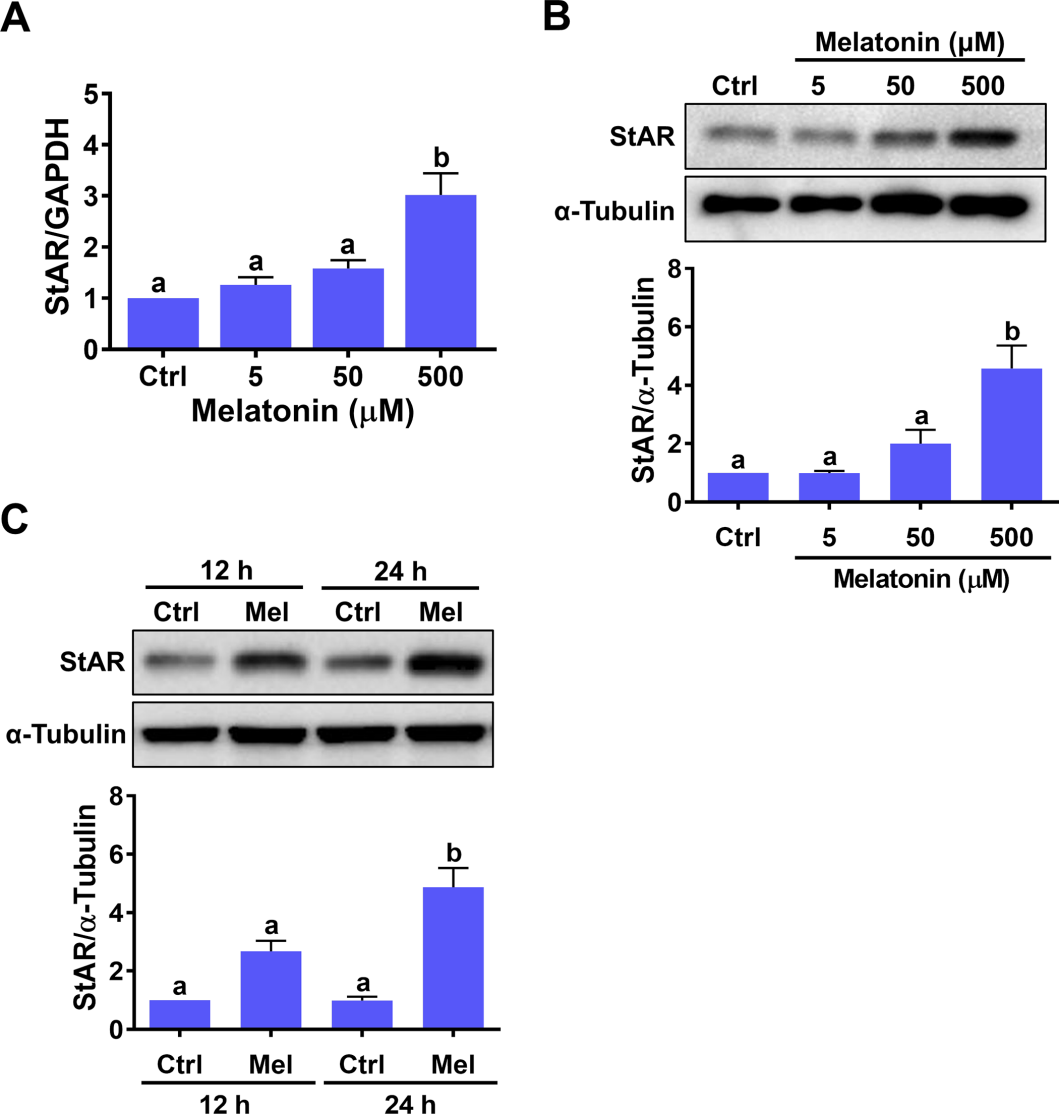
**Melatonin stimulates StAR expression in primary human granulosa-lutein cells.** Human granulosa-lutein (hGL) cells were treated with different concentrations of melatonin (Mel) for 24 h, and StAR mRNA (**A**) and protein (**B**) levels were examined by RT-qPCR and western blot, respectively. (**C**) Cells were treated with 500 μM melatonin for 12 and 24 h, and StAR protein levels were examined by western blot. Results are expressed as the mean ± SEM of 4 independent experiments. Values without a common letter are significantly different (*p* < 0.05).

To examine the effect of melatonin on StAR expression, hGL cells isolated from follicular aspirates of women undergoing oocyte retrieval during IVF treatment were treated with different concentrations of melatonin for 24 h. While 5 or 50 μM melatonin had no significant effects, StAR mRNA levels were significantly upregulated by exposure to 500 μM melatonin (Figure 1A). Western blot results confirmed the stimulatory effects of melatonin on StAR at the protein level (Figure 1B). Time-course expression experiments revealed that 12 h melatonin treatment caused a slight, non-significant upregulation of StAR protein levels, while significant upregulation was observed after 24 h of treatment (Figure 1C).

### Melatonin-induced StAR expression is mediated by MT1 and MT2 receptors

**Figure 2:**
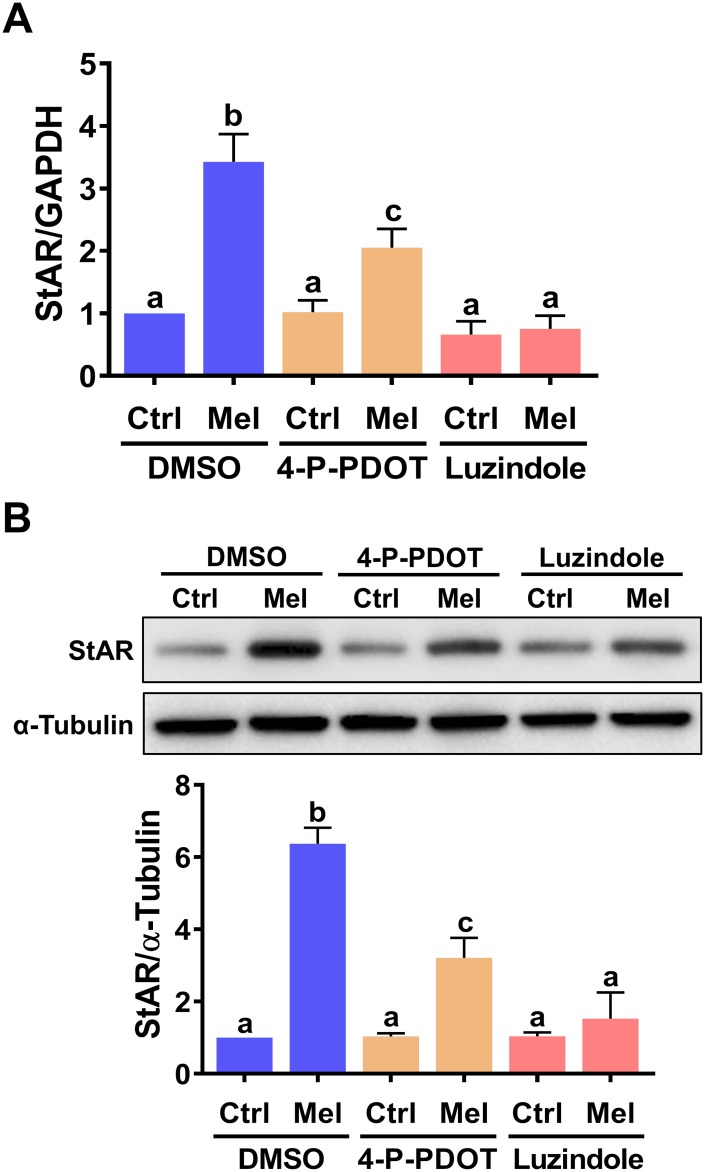
**MT1 andMT2 melatonin receptors mediate melatonin-induced StAR expression in primary hGL cells.** Cells were pre-treated with vehicle control (DMSO), 10 μM 4-P-PDOT, or 10 μM luzindole for 30 min and then exposed to 500 μM melatonin for 24 h. StAR mRNA (**A**) and protein (**B**) levels were examined by RT-qPCR and western blot, respectively. Results are expressed as the mean ± SEM of 4 independent experiments. Values without a common letter are significantly different (*p* < 0.05).

To identify the cellular receptor(s) involved in melatonin-induced StAR expression in hGL cells, two melatonin receptor antagonists, 4-P-PDOT (MT2-selective) and luzindole (MT1/MT2-nonselective), were tested [29]. As shown in Figure 2A, none of these inhibitors affected basal StAR mRNA levels. However, in the presence of melatonin, StAR mRNA upregulation was partially inhibited by pre-treatment with 4-P-PDOT, and abolished by pre-treatment with luzindole. Furthermore, western blot analyses showed that these antagonists also reduced StAR protein expression (Figure 2B). These results indicate that both MT1 and MT2 mediate melatonin-induced upregulation of StAR expression in hGL cells.

### PI3K/AKT signaling mediates melatonin-induced StAR expression

**Figure 3:**
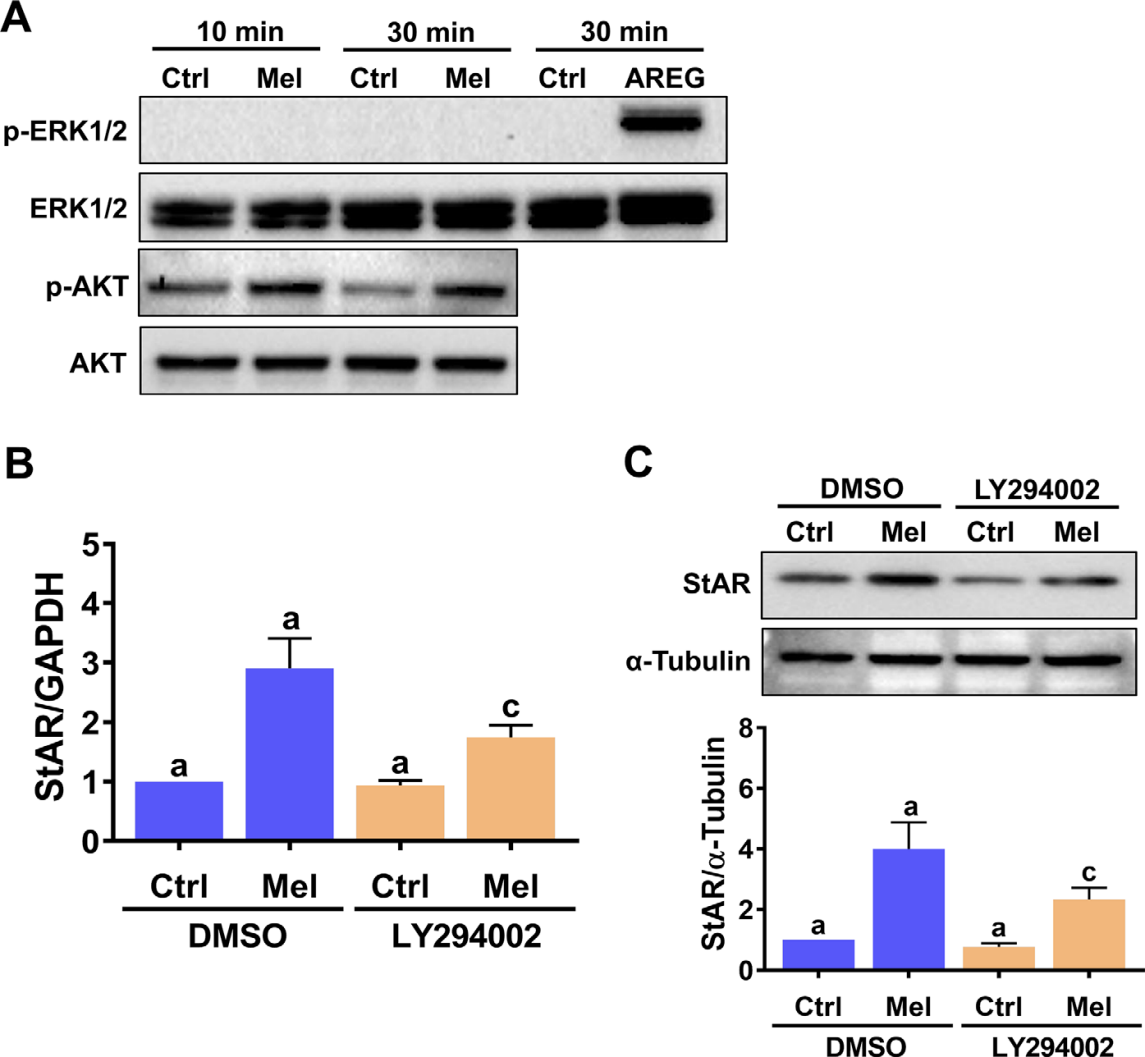
**Melatonin-induced StAR expression is partly mediated by PI3K/AKT activation.** (**A**) hGL cells were treated with 500 μM melatonin for 10 or 30 min, and both total and phosphorylated ERK1/2 and AKT expression was determined by western blot. Cells treated with 100 ng/mL amphiregulin (AREG) were used as positive control for ERK1/2 phosphorylation. (**B**, **C**) hGL cells were pre-treated with vehicle control (DMSO) or 10 μM LY294002 for 30 min and then exposed to 500 μM melatonin for 24 h. StAR mRNA (**B**) and protein (**C**) levels were examined by RT-qPCR and western blot, respectively. Results are expressed as the mean ± SEM of 3 independent experiments. Values without a common letter are significantly different (*p* < 0.05).

Upon binding to MT1/MT2 receptors, melatonin can activate the MEK/ERK1/2 and PI3K/AKT signaling pathways in a cell type-dependent manner [30]. Therefore, we examined the effect of melatonin on the activity of these two signaling pathways in hGL cells. As shown in Figure 3A, melatonin treatment increased phospho-AKT levels, indicating PI3K/AKT activation, but did not elicit ERK1/2 activation. We used amphiregulin as a positive control, since we have shown that it can activate ERK1/2 signaling in hGL cells [31]. Next, we tested a specific PI3K inhibitor, LY294002, to further determine whether PI3K is required for melatonin-induced upregulation of StAR expression. As shown in Figure 3B and 3C, pre-treatment with LY294002 partially attenuated melatonin-induced upregulation of StAR mRNA and protein levels. These results indicate that activation of the PI3K/AKT signaling pathway is involved in melatonin-induced StAR expression in hGL cells.

### StAR expression is required for melatonin-stimulated P4 production in hGL cells

**Figure 4:**
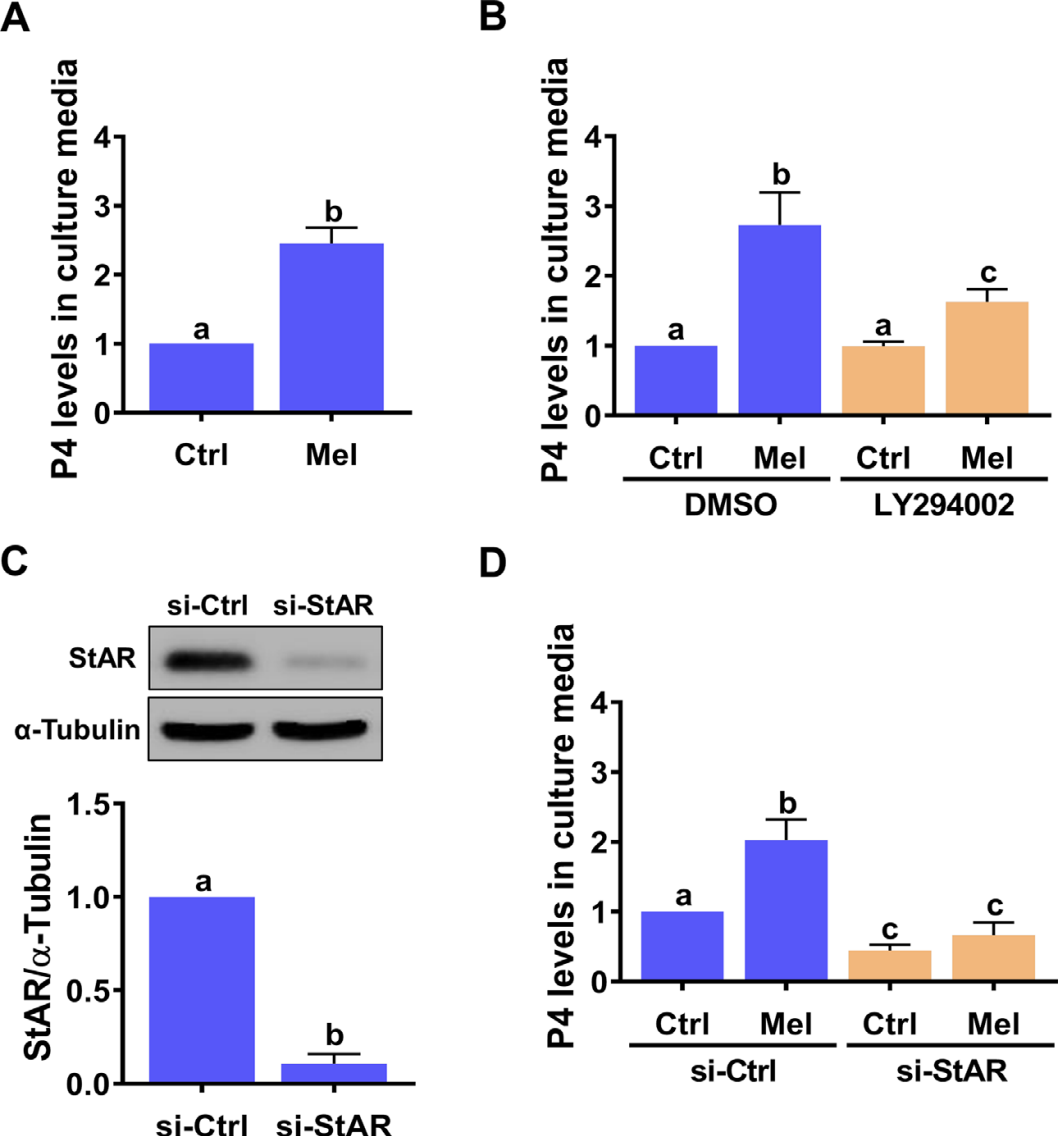
**StAR is required for melatonin-induced P4 production in primary hGL cells.** (**A**) Cells were treated with 500 μM melatonin for 24 h, and P4 levels in culture media examined using ELISA. (**B**) Cells were pre-treated with vehicle control (DMSO) or 10 μM LY294002 for 30 min and then exposed to 500 μM melatonin for 24 h. P4 levels in culture media were examined using ELISA. (**C**, **D**) Cells were transfected with 50 nM control siRNA (si-Ctrl) or StAR siRNA (si-StAR) for 48 h. (**C**) StAR siRNA knockdown efficiency was examined by western blot. (**D**) P4 levels in the culture media of si-Ctrl and si-StAR transfected hGL cells treated with 500 μM melatonin for 24 h were examined using ELISA. Results are expressed as the mean ± SEM of 4 independent experiments. Values without a common letter are significantly different (*p* < 0.05).

Given the critical role of StAR in the regulation of P4 production, we examined the effect of melatonin on the production of P4 in hGL cells. ELISA showed that hGL cells stimulated with melatonin released P4 into the culture medium (Figure 4A), and that this effect was attenuated by inhibition of the PI3K/AKT signaling pathway (Figure 4B). To directly examine the requirement of StAR for melatonin-stimulated P4 production, a siRNA-based gene silencing approach was used to knockdown StAR expression. Transfection of hGL cells with StAR siRNA for 48 h significantly downregulated endogenous StAR protein levels (Figure 4C), downregulated basal P4 expression, and abolished melatonin-stimulated P4 secretion (Figure 4D).

### Melatonin levels in follicular fluid are positively correlated with serum P4 levels

**Figure 5:**
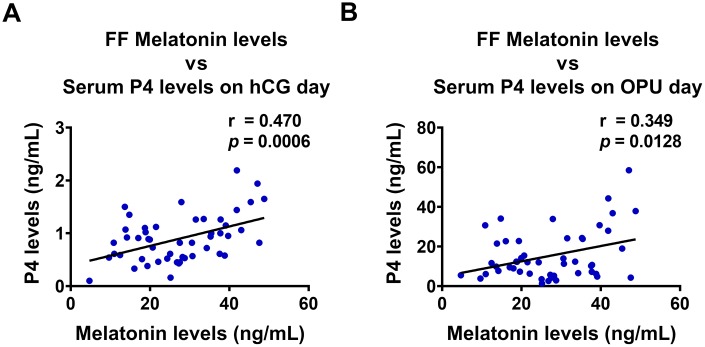
**Melatonin levels in follicular fluid are positively correlated with P4 levels in serum.** Follicular fluid (FF) melatonin levels and serum P4 levels were examined using ELISA (n = 50), and Pearson’s correlation analysis was performed to assess their relationship. FF melatonin levels were positively correlated with serum P4 levels both on (**A**) hCG administration day, and (**B**) oocyte pick-up (OPU) day.

Follicular fluid provides a critically important microenvironment for the development of the ovarian follicle and the oocyte. Therefore, we examined the relationship between follicular fluid melatonin and serum P4 levels in clinical samples from 50 IVF patients. Interestingly, follicular melatonin levels were positively correlated with serum P4 levels both on hCG administration day (r = 0.470, *p* = 0.0006) and on the day of oocyte pick-up (OPU) (r = 0.349, *p* = 0.0128) (Figures 5A and 5B). These results strongly support the *in vitro* stimulatory effect of melatonin on P4 production in the human ovary.

## DISCUSSION

Previous studies have shown that melatonin is able to stimulate the production of P4 in the ovary [22, 23, 25, 32, 33]. Unlike animal studies, in which granulosa cells at various differentiation stages can be obtained and used to examine hormonal actions, nearly all human studies are restricted to the use of highly differentiated granulosa cells obtained from women undergoing IVF treatment. Due in part to this constrain, and despite available evidence, the effect of melatonin on P4 production by hGL cells remains controversial. A few studies showed that exposure to melatonin does not alter basal P4 production in hGL cells [16, 34, 35]. However, both stimulatory [21, 36] and inhibitory [37, 38] effects of melatonin on basal P4 production have also been reported. Research showed that during IVF treatment stimulation with high doses of gonadotrophins prior to oocyte retrieval results in low response to gonadotrophins on the first day of hGL cell culture, due to ligand-binding-induced downregulation of gonadotrophin receptors. However, more prolonged hGL culturing can increase responsiveness to gonadotrophins by restoring the expression of their receptors [39]. Similarly, we found that protein expression of MT1 and MT2 was barely detectable on the first day of culture, but was restored after 5 days of culture (Supplementary Figure 1). This observation is supported by previous studies [30]. Therefore, in our system, hGL cells were cultured for 5 days before being used in experiments. Although detailed experimental conditions were not provided by some previous studies, we believe that culture conditions strongly affect the biological functions of melatonin in hGL cells *in vitro*, and may explain why P4 production was not affected by melatonin treatment in previous investigations.

In contrast with our results, two previous studies showed that melatonin treatment inhibited basal P4 production in hGL cells [37, 38]. Unlike our study, which used hGL cells derived from follicular aspirates of women undergoing oocyte retrieval during IVF treatment, in one such study hGL cells were obtained from women undergoing ovariectomy for cancer of the uterus [37]. This significant methodological difference might explain the contradictory results. In the other referred study, low concentrations of melatonin did not affect basal P4 production, while treatment with 1 mM melatonin for 2 days significantly decreased P4 synthesis [38]. Since mitogenic and anti-apoptotic effects of melatonin on granulosa cells have been reported [28], dose- and time-dependent cellular responses might account for such reduction. Moreover, in our study P4 levels in culture media were measured and normalized against corresponding protein concentrations. It is unclear whether that same approach was taken in the referred study [38], and this factor may further account for the observed discrepancy.

In humans, melatonin signals through two cellular receptors, MT1 and MT2, which mediate the activation of different intracellular signaling pathways [30]. As both MT1 and MT2 are expressed in hGL cells, we used two different antagonists, 4-P-PDOT (MT2-selective) and luzindole (MT1/MT2-non-selective), to delineate the involvement of MT1 and MT2 in melatonin-induced StAR expression. Our results showed that melatonin-induced StAR expression was partially attenuated by 4-P-PDOT, but completely abolished by luzindole. These results indicate that both MT1 and MT2 are involved in melatonin-induced StAR upregulation. Consistent with our results, two recent studies using similar approaches demonstrated that melatonin-induced upregulation of StAR expression and P4 production are partially attenuated by 4-P-PDOT treatment but abolished by treatment of luzindole in the corpus luteum of pregnant sows and in bovine theca cells [25, 26]. Similarly, both MT1 and MT2 are involved in melatonin-induced P4 production in bovine granulosa cells [32, 33, 40]. Taken together, these results indicate that melatonin-induced StAR expression and P4 production are mediated by both MT1 and MT2.

Multiple signaling pathways, including PI3K/AKT and ERK1/2, as well as several transcription factors, are involved in the regulation of steroidogenesis and StAR expression in ovarian follicles [24] Several studies showed also that PI3K/AKT and ERK1/2 signaling can be activated by melatonin in different cell types [30]. Our previous study has shown that activation of ERK1/2, but not PI3K/AKT, signaling is required for amphiregulin-induced StAR expression in hGL cells [31]. Interestingly, in the current study we showed that melatonin activated the PI3K/AKT signaling pathway without affecting the activation of ERK1/2 in hGL cells, and PI3K/AKT activation was required for the induction of StAR expression. It is generally considered that theca cells are the major site of ovarian androgen production, while granulosa cells are the main source of P4 and estradiol [41, 42]. However, a recent study also detected StAR expression and P4 production in bovine theca cells, which could be blocked by inhibition of PI3K/AKT signaling by LY294002 or wortmannin [26]. Along with our results, these findings demonstrate the involvement of the PI3K/AKT signaling pathway in melatonin-induced StAR expression and P4 production in the ovarian follicle. Moreover, it was reported that activation of PI3K/AKT signaling is also required for FSH- and TGF-β1-stimulated StAR expression in rat granulosa cells [43]. However, since inhibition of PI3K/AKT signaling did not completely block melatonin-induced StAR expression, the involvement of other signaling pathways seems plausible. Therefore, more studies will be needed to delineate the molecular mechanisms that mediate the stimulatory effect of melatonin on StAR expression in hGL cells.

Melatonin levels in human follicular fluid increase as the follicle grows [44]. High concentration of melatonin in follicular fluid has been suggested to protect hGL cells from oxidative stress, allowing them to sustain P4 production [35]. In addition, the antioxidant activity of melatonin was shown to favor oocyte maturation and ovulation [28, 45]. These findings reveal important roles of follicular melatonin in the regulation of female reproductive functions. In the present study, we showed that melatonin levels in follicular fluid were positively correlated with P4 levels in sera collected both on hCG administration and OPU days. Along with the data obtained from *in vitro* experiments, these clinical results confirmed the stimulatory effect of melatonin on StAR expression and P4 production in the human ovary.

In summary, the present study demonstrates that short exposure (24h) to melatonin stimulates StAR expression in hGL cells, resulting in P4 production. These effects are mediated by MT1 and MT2 receptors, and are partially dependent on activation of the PI3K/AKT signaling pathway. Moreover, melatonin levels in follicular fluid are positively correlated with P4 levels in serum. These results suggest a time-and dose-dependent physiological role for melatonin in the regulation of StAR expression and P4 production in hGL cells, and might help develop new strategies for the treatment of clinical infertility.

## MATERIALS AND METHODS

### Antibodies and reagents

Polyclonal anti-StAR antibody was obtained from Santa Cruz Biotechnology (Shanghai, China). Monoclonal anti-α-tubulin antibody was obtained from CMCTAG (Shanghai, China). Monoclonal anti-phospho-ERK1/2 (Thr^202^/Tyr^204^) and polyclonal anti-ERK1/2, anti-phospho-AKT (Ser^473^), and anti-AKT antibodies were obtained from Cell Signaling Technology (Shanghai, China). Polyclonal anti-MT1 was obtained from Bioworld Technology (Nanjing, China). Polyclonal anti-MT2 was obtained from Abcam (Shanghai, China). Horseradish peroxidase-conjugated goat anti-rabbit and goat anti-mouse IgGs were obtained from Bio-Rad Laboratories (Shanghai, China). Melatonin, 4-P-PDOT, luzindole, and LY294002 were obtained from Sigma-Aldrich Corp (Shanghai, China).

### Human serum and follicular fluid samples

The study received institutional approval and was carried out in accordance with the guidelines from the Zhengzhou University Research Ethics Board. Human serum and follicular fluid samples were obtained from 50 infertile women during IVF treatment. All patients were between the ages of 20 and 35 and had normal menstrual cycles. Causes of infertility were tubal obstruction or male infertility. Patients with polycystic ovarian syndrome, endometriosis, diminished ovarian reserve, chromosome abnormality, or hydrosalpinx were excluded from the study. All patients were treated with a standard long protocol. At the mid-luteal phase, the gonadotropin-releasing hormone (GnRH) agonist triptorelin (0.1 mg) (Ipsen Pharma Biotech, France), was administered subcutaneously daily. Approximately 14 days after GnRH agonist injection was started, recombinant FSH (Gonal-F; Merck, Germany) was administered daily at a dosage of 150–300 IU. When at least three follicles had reached 18 mm, hCG (10,000 IU, Livzon, Zhuhai, China) was injected. Oocyte retrieval was scheduled approximately 34–36 h after hCG injection by transvaginal ultrasound-guided follicular aspiration. Blood samples were obtained by venipuncture. After collection, serum was stored at −80 °C until further analysis. The follicular fluid was collected when the oocytes were retrieved. Only the first follicular fluid aspirate without blood or flushing solution was used for analysis. After 10 min of centrifugation at 1200 rpm, the supernatant was stored at −80 °C until further analysis.

### Primary culture of human granulosa-lutein (hGL) cells

Primary hGL cells were purified by density centrifugation from follicular aspirates collected from women undergoing oocyte retrieval as previously described [46, 47]. Cells were cultured in a humidified atmosphere containing 5% CO_2_ and 95% air at 37°C in Dulbecco’s Modified Eagle Medium/nutrient mixture F-12 Ham medium (DMEM/F-12; Gibco, Shanghai, China) supplemented with 10% charcoal/dextran-treated FBS (HyClone, Shanghai, China), 100 U/mL of penicillin, and 100 μg/mL of streptomycin sulfate (Boster, Wuhan, China). For melatonin stimulation experiments, cells were cultured in 12-well plates at a density of 5 × 10^4^ cells/cm^2^ with 1 mL of culture medium for 5 days. All treatments were performed in medium containing 0.5% charcoal/dextran-treated FBS.

### Reverse transcription quantitative real-time PCR (RT-qPCR)

Total RNA was extracted with the RNeasy Plus Mini Kit (QIAGEN, Shanghai, China) according to the manufacturer’s instructions. RNA (2 μg) was reverse-transcribed into first-strand cDNA with the High-Capacity cDNA Reverse Transcription Kit (Applied Biosystems, Shanghai, China). Each 20 μL RT-qPCR reaction contained 1X SYBR Green PCR Master Mix (Applied Biosystems), 60 ng of cDNA, and 250 nM of each specific primer. The primers used were 5′-AAA CTT ACG TGG CTA CTC AGC ATC-3′ (sense) and 5′-GAC CTG GTT GAT GAT GCT CTT G-3′ (antisense) for steroidogenic acute regulatory protein (StAR) and 5′-GAG TCA ACG GAT TTG GTC GT-3′ (sense) and 5′-GAC AAG CTT CCC GTT CTC AG-3′ (antisense) for GAPDH. RT-qPCR was performed on an Applied Biosystems QuantStudio 12K Flex system equipped with 96-well optical reaction plates. The specificity of each assay was validated by melting curve analysis and by agarose gel electrophoresis of the PCR products. RT-qPCR experiments were run in triplicate, and a mean value was used to determine the mRNA levels. Water and mRNA without RT enzyme were used as negative controls. Relative quantification of mRNA levels was performed using the comparative Ct method with GAPDH as the reference gene, using the formula 2^–ΔΔCt^.

### Western blotting

Cells were lysed in cell lysis buffer (Cell Signaling Technology). Equal amounts of protein were separated by SDS polyacrylamide gel electrophoresis and transferred onto PVDF membranes. After 1 h blocking with 5% non-fat dry milk in Tris-buffered saline (TBS), the membranes were incubated overnight at 4 °C with primary antibodies diluted in 5% non-fat milk/TBS. Following primary antibody incubation, the membranes were incubated with appropriate HRP-conjugated secondary antibodies. Immunoreactive bands were detected using an enhanced chemiluminescent substrate (Bio-Rad Laboratories (Shanghai, China), and imaged with a ChemiDoc MP Imager (Bio-Rad Laboratories).

### Small interfering RNA (siRNA) transfection

To knock down endogenous StAR, cells were transfected with 50 nM ON-TARGETplus SMARTpool StAR siRNA (Dharmacon, Shanghai, China) using Lipofectamine RNAiMAX (Invitrogen, Shanghai, China). The siCONTROL NON-TARGETING pool siRNA (Dharmacon) was used as the transfection control. Knockdown efficiency was examined using western blot.

### Measurement of melatonin and progesterone

Melatonin levels in follicular fluids were measured using an enzyme-linked immunosorbent assay (ELISA Kit, Abcam, Shanghai, China) in accordance with the manufacturer’s protocol. Serum progesterone (P4) levels were measured using an ELISA Kit (Cayman Chemical, Shanghai, China) as per the manufacturer’s instructions. P4 levels in culture media were normalized to protein concentrations from corresponding cell lysates. For each treatment, normalized culture media P4 levels were expressed as relative values in comparison to control treatment.

### Statistical analysis

Results are presented as the mean ± SEM of at least three independent experiments. All statistical analyses were conducted on PRISM software. For experiments involving only two groups, data were analyzed by t test. Multiple comparisons were made using one-way ANOVA followed by Tukey’s multiple comparison test. Statistical significance was defined as *p* < 0.05.

## Supplementary Material

Supplementary Figure
