# Supplementary material for: Melatonin induces progesterone production in human granulosa-lutein cells through upregulation of StAR expression
Source: Aging (Albany NY). 2019 Oct 16;11(20):9013–24. doi: 10.18632/aging.102367 (PMC6834401; doi:10.18632/aging.102367)
Supplement: Supplementary Figure [file aging-11-102367-s001.pdf]

SUPPLEMENTARY FIGURE

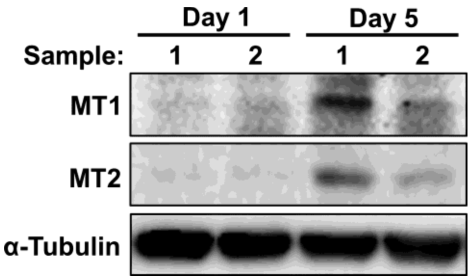

Supplementary Figure 1. Expression of melatonin receptors in primary human granulosa-lutein (hGL) cells. MT1 and MT2 expression was examined by western blot using total hGL cell lysates (n = 2) collected after 1 or 5 days of culture.
